# Emergence of new red-shifted carbon nanotube photoluminescence based on proximal doped-site design

**DOI:** 10.1038/srep28393

**Published:** 2016-06-27

**Authors:** Tomohiro Shiraki, Tomonari Shiraishi, Gergely Juhász, Naotoshi Nakashima

**Affiliations:** 1 Department of Applied Chemistry, Graduate School of Engineering, Kyushu University, 744 Motooka, Nishi-ku, Fukuoka 819-0395, Japan; 2 International Institute for Carbon-Neutral Energy Research (WPI-I2CNER), Kyushu University, 744 Motooka, Nishi-ku, Fukuoka, Japan; 3 Department of Chemistry, Graduate School of Science, Tokyo Institute of Technology, Ookayama, Meguro-ku, Tokyo, 152-8550, Japan

## Abstract

Single-walled carbon nanotubes (SWNTs) show unique photoluminescence (PL) in the near-infrared (NIR) region. Here we propose a concept based on the proximal modification in local covalent functionalization of SWNTs. Quantum mechanical simulations reveal that the SWNT band gap changes specifically based on the proximal doped-site design. Thus, we synthesize newly-designed bisdiazonium molecules and conduct local fucntionalisation of SWNTs. Consequently, new red-shifted PL (*E*_11_^2*^) from the bisdiazonium-modified SWNTs with (6, 5) chirality is recognized around 1250 nm with over ~270 nm Stokes shift from the PL of the pristine SWNTs and the PL wavelengths are shifted depending on the methylene spacer lengths of the modifiers. The present study revealed that SWNT PL modulation is enable by close-proximity-local covalent modification, which is highly important for fundamental understanding of intrinsic SWNT PL properties as well as exciton engineering–based applications including photonic devices and (bio)imaging/sensing.

Single-walled carbon nanotubes (SWNTs), which consist of a rolled-up single graphene sheet, have remarkable properties based on their unique one-dimensional (1D) nanostructures^1^. In particular, the absorption and photoluminescence (PL) of the SWNTs arise in the near infra-red (NIR) region by the Van Hove singularity^2^. Based on the advantage of the NIR PL in the high transparency for biological tissues and in the telecommunication wavelengths, applications for imaging, sensing and optical devices have been widely studied^3^,^4^,^5^,^6^,^7^,^8^. A fundamental drawback of the SWNT materials, however, is the low quantum yield (φ < 1%)^9^ due to i) optically-forbidden transition states for dark exciton that exist at the lower energy levels than the optically-allowed transition states for the bright exciton^10^, and ii) exciton quenching through collision with defect sites^11^ or with other excitons^12^ that can frequently occur owing to the 1D-confined structure. Recently, a new method to dramatically enhance the φ values has been reported, which arises from a very limited amount of chemical modification through oxygen atom doping^13^ and *sp*^3^ defect doping^14^,^15^,^16^ on the *sp*^2^ network of the SWNTs. The resultant locally-functionalized SWNTs (local-f-SWNTs) show new emission (*E*_11_^*^) with high efficiency by an exciton trapping mechanism at the emissive doped sites.

Very recently, we successfully determined the electronic states of the oxygen-doped (n,m)SWNTs (O-doped (n,m)SWNTs) on the basis of an *in situ* PL spectroelectrochemical analysis^17^,^18^,^19^,^20^. Importantly, the observed HOMO and LUMO level shifts depended on the O-doped structures^21^. In the *sp*^3^ defect doping system, substituents on the introduced aryl groups provided a band gap variation^14^. In this study, we present a new concept for the SWNT PL modulation based on the doping-induced energy level shifting in which proximal chemical modification using bisdiazonium compounds is adopted for the multipoint modification in the local-f-SWNTs (Fig. 1a,b). The key feature is readily applicable to a series of modifiers bearing multi-diazonium groups for further PL modulation based on this concept. To the best of our knowledge, this is the first report describing the proximal modification-induced PL generation and its modulation based on molecular designs for local covalent modification of the SWNTs.

## Results and Discussion

**Figure 1:**
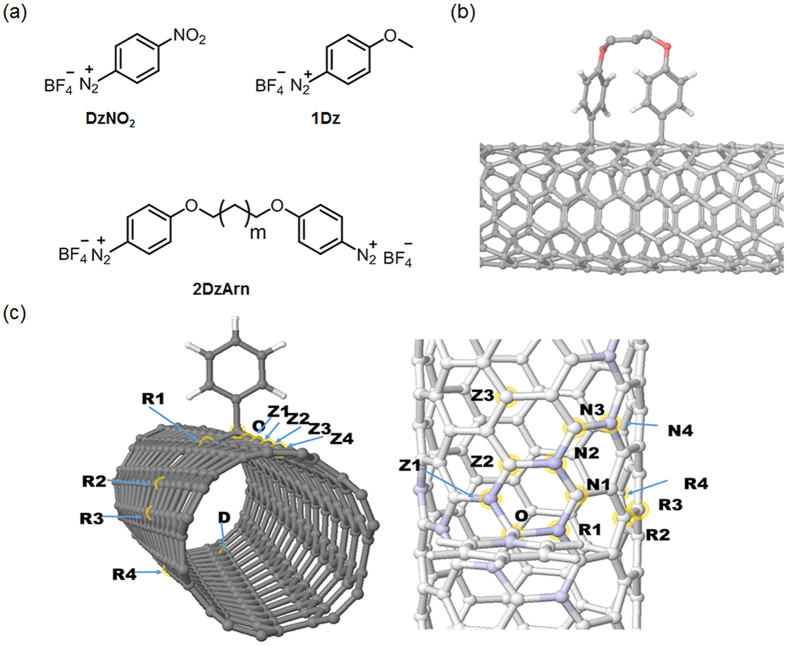
Modifiers and the functionalized SWNT models. (**a**) Chemical structures of diazonium compounds. (**b**) Schematic image of multipoint modification of SWNTs using 2DzArn to introduce proximal *sp*^3^ defects. (**c**) Calculated model structures showing the relative position of the doped sites (left) and position of doped sites near O (right). The spin-density around O in a single doped site is indicated by shades of blue.

To prove our concept, quantum mechanical calculations were carried out. Previously, experimental results of the local-f-SWNTs showed that the PL enhancement can only be observed under mild doping conditions, when the average distance between the doped sites is at least 20 nm^13^,^14^. One may conclude that there is a strong electronic interaction between doped sites even over large distances, and therefore a low concentration of doping is needed to avoid quenching the excitons. When a nanotube is doped with an *sp*^3^ site, a half field orbital (SOMO) is introduced to the band gap, indicating the radical character of the doped site. With two-*sp*^3^ doping sites, two new energy levels appear in the band gap with almost identical energies, therefore, close to a zero HOMO-LUMO gap if there is no electronic interaction between these sites. Calculations on the (6,5) tube using the Density Functional Tight Binding (DFTB) method showed that the interaction between the doping sites is rare even over short distances. Figure 1c and Table 1 summarize the model structures, their relative stability and calculated HOMO-LUMO gaps. We investigated various dopant positions where the first dopant was placed on position **O** (see in Fig. 1c) and a second at a distant position (**D**, *d*(**OD**) = 2.06 nm) along the axis (**Z1**…**Z4**), around the tube (**R1**…**R4**) and in a close proximity to **O** (**N1**…**N4**). As shown in Table 1, in almost all the cases, the HOMO-LUMO gap remained smaller than 0.03 eV with the only exceptions of **Z1** (0.94 eV), **R1** (0.95 eV), **R3** (0.58 eV), **N2** (0.95 eV) and **N4** (0.74 eV). The calculated HOMO-LUMO gap of the pristine (6,5) tube is 0.95 eV, therefore the energies at **R3** and **N4** correspond to 0.21 eV and 0.37 eV changes, respectively. This suggest the possibility of new *E*_11_^*^-type peaks in the optical spectrum. We would like to emphasize that such a small HOMO-LUMO gap can be observed even in close geometries like **Z2** or **N1**, in which cases the distances between the two doped sites are only 0.251 and 0.254 nm, respectively. The position of these sites where the interaction was observed correlate very well with the spin density around **O** when only a single dopant is used (see Fig. 1c and Table 1), proving that the coupling between the doped sites is a resonance effect. Considering the selectivity of the doping process, when the first aryl group is attached, the resultant unpaired electron density makes these sites on the tube wall more active, therefore, we expect that the second dopant shows a preference for these resonant sites. This result encouraged us to use our approach of multipoint modification on the SWNTs for new PL induction with a different emission wavelength.

**Figure 2:**
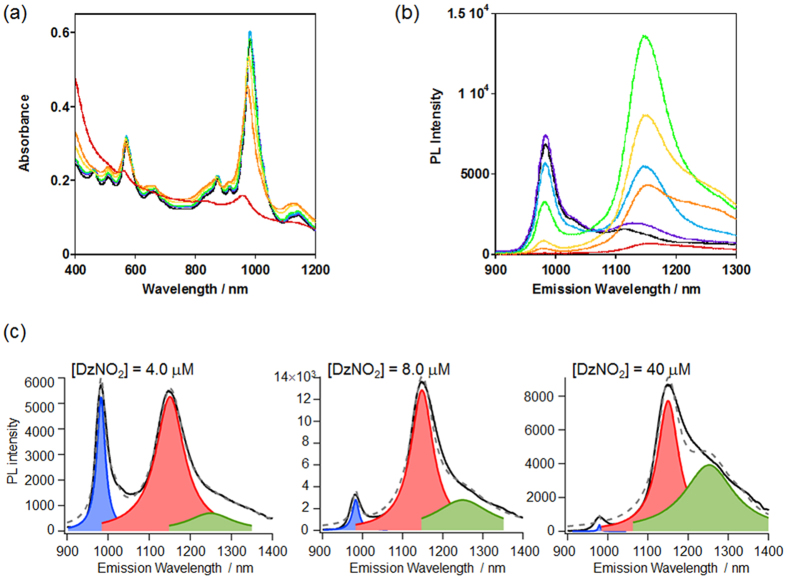
Spectral changes in the SWNTs modified with DzNO_2_. (**a**) Vis/NIR and (**b**) PL spectra of SWNT/DzNO_2_ prepared by varying DzNO_2_ concentrations with 0 (black), 0.40 (violet), 2.0 (light blue), 4.0 (lime), 40 (yellow), 80 (orange) and 200 μM (red) in D_2_O. λ_ex_ = 570 nm. (**c**) Representative deconvoluted PL spectra of the SWNT/DzNO_2_.

As a preliminary experiment, we increased the reactant concentrations to raise the density of the doped *sp*^3^ defect sites on the tubes by using the DzNO_2_ tethering one reactive diazonium to obtain DzNO_2_-modifed SWNTs (SWNT/DzNO_2_), and monitored their PL spectral changes. In this study, the (6,5)-enriched CoMoCat-SWNTs were used. As shown in Fig. 2a,b, the reaction using 4.0 μM DzNO_2_ provided an *E*_11_^*^ emission band at 1146 nm on the modified (6,5)SWNTs, which is red-shifted from the *E*_11_ PL (980 nm) of non-modified (6,5)tubes, although almost no change in the absorption spectra was recognized before and after the reaction owing to the limited amount of the chemical modification, which is consistent with the results for previously-reported local-f-SWNTs^13^,^14^,^15^,^16^. We observed an increase in the PL intensity at around 1250 nm upon the addition of DzNO_2_, indicating that longer wavelength emission than *E*_11_^*^ appears in the reaction mixtures with higher DzNO_2_ concentrations, which is clearly distinguishable from the deconvoluted spectra as shown in Fig. 2c. Emergence of the *E*_11_^*^ emission arises from the dissociation of a degenerate energy band through symmetry breaking of the SWNT structures due to the chemical modification. Thus, a possible explanation for the new PL generation at 1250 nm would be the adjacent attachment of the aryl groups on the tubes at higher DzNO_2_ concentrations, which relates to the theoretical considerations described above. In this case, however, the total PL intensity decreased with respect to the increase in the DzNO_2_ concentrations because of severe disruption of the inherent *sp*^2^ and π-conjugated tube structure, as clearly observed in the 40–200 μM conditions (Fig. 2b).

**Figure 3:**
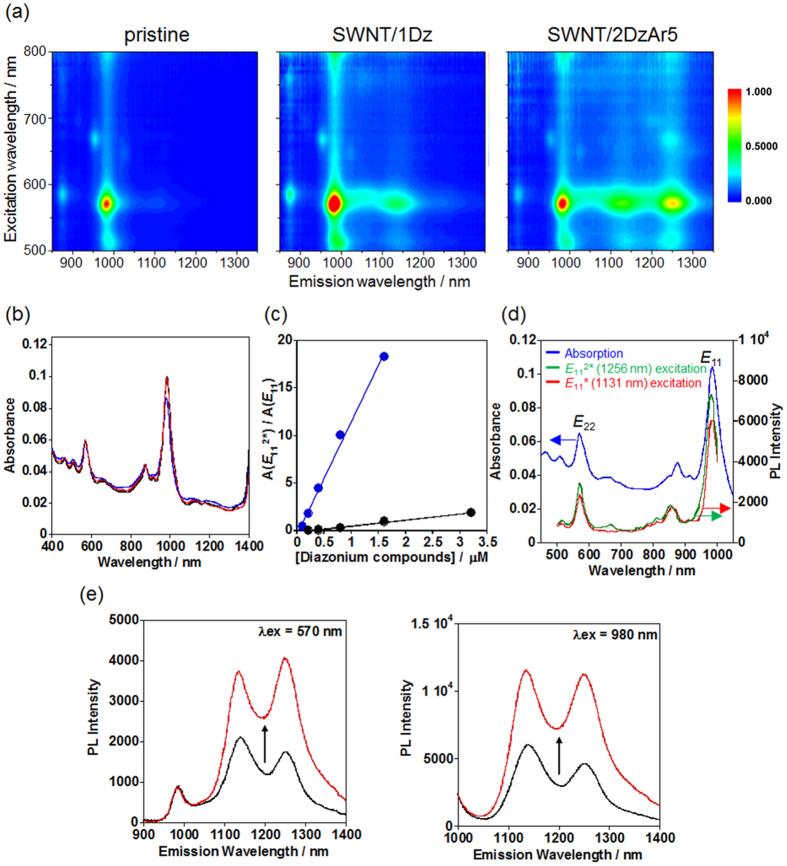
Optical properties of the SWNT/2DzAr5. (**a**) 2D PL maps of pristine SWNT, SWNT/1Dz, and SWNT/2DzAr5 and (**b**) vis/NIR absorption spectra of pristine SWNTs (black), SWNT/1Dz (blue), and SWNT/2DzAr5 (red) in D_2_O. [1Dz] = 0.40 μM and [2DzAr5] = 0.20 μM are used for the chemical modification. (**c**) PL area ratios of *E*_11_^2^* to *E*_11_ (A(*E*_11_^2^*)/A(*E*_11_)) as a function of the diazonium concentrations for SWNT/2DzAr5 (blue dot) and SWNT/1Dz (black dot). (**d**) Excitation spectra for *E*_11_^2^* (green) and *E*_11_* (red) and vis/NIR absorption spectrum (blue) of SWNT/2DzAr5 in D_2_O. [2DzAr5] = 0.40 μM was used for the chemical modification. (**e**) PL spectra of SWNT/2DzAr5 before (black) and after (red) dialysis against a 0.2 wt% (5.7 mM) SDBS solution: λ_ex_ = 570 nm and 980 nm.

On the basis of the theoretical calculations and the preliminary experiments, we newly designed SWNT modifiers that are bisdiazonium compounds with a methylene linker (2DzArn, n = 3, 5 and 9) for the selective proximal modification of the SWNTs, in which the local density of the doped *sp*^3^ defects at the modified sites can be tunable without excess any addition of the reactants. The 2DzArn (n = 3, 5 and 9) were synthesized by the Williamson ester synthesis from 4-hydroxyacetanilide and dibromoalkanes followed by hydrolysis of the amide group and diazotization (for details, see Methods section and Fig. S1). As a monodiazonium control, 4-methoxybenzenediazonium tetrafluoroborate (1Dz) was used. As shown in Fig. 3a, the 2D-PL mapping of the 2DzAr5-functionalized SWNTs (SWNT/2DzAr5) clearly shows a new peak (*E*_11_^2*^) at 1256 nm, which has hitherto never been observed for the SWNT/1Dz. The *E*_11_^*^ peak at 1129 nm appeared for both the SWNT/1Dz and SWNT/2DzAr5, which is PL from the doped site with the monodiazonium modification. The observed *E*_11_^2^* and *E*_11_*peaks are reproducible and the intensities were not changed during the optical measurements under light irradiation probably due to the very limited amount of doped site formation on the tubes. In the vis/NIR absorption shown in Fig. 3b, the spectra of pristine SWNTs and SWNT/2DrAr5 were almost identical, indicating a very low degree of chemical modification on the tubes, which agreed with the results for the previously reported local-f-SWNTs^13^,^14^,^15^,^16^. When the amount of added 1Dz increased, the *E*_11_^2^* peak was barely observed (Fig. S2a). In stark contrast, the *E*_11_^2^* peak is significant and the intensity was increased for SWNT/2DzAr5 prepared by the further addition of 2DzAr5 owing to the increase in the number of the doped sites created by 2DzAr5 (Fig. S2b), which is clearly recognized by the remarkable increase in the integrated area ratio of *E*_11_^2^* to *E*_11_* for SWNT/2DzAr5 (18.3) compared to that for SWNT/1D (1.87) when the concentrations based on the diazonium unit were 3.2 μM, as shown in Fig. 3c. The excitation spectra of *E*_11_^2^* and *E*_11_* of the SWNT/2DzAr5 sample are described in Fig. 3d, in which the spectra almost overlapped with the absorption spectrum and both of the PLs were similarly induced by excitation using *E*_11_ and *E*_22_, respectively, showing that excitons from the non-modified sites should contribute to the emissions. It shows that the emission mechanism of *E*_11_^2^* should arise from the exciton trapping process at the doped site like that of the *E*_11_*. Accordingly, we conclude that the newly-observed *E*_11_^2*^ at 1256 nm originates from the local covalent modification by the 2DzAr5 on the tubes. Very recently, Wei *et al*. have reported that the naturally-oxidized (5,4)SWNTs showed a small PL peak at 1096 nm which is lower energy emission than *E*_11_ (829 nm) and *E*_11_* (968 nm) and suggested that the observed emission may be due to epoxy structure formation at the oxygen-doped sites^22^. The result also indicates that the doped-chemical structures on the SWNTs dominate the resulting emission properties. Thus, our approach based on structural design of the doped sites by using molecularly-designed modifiers would be promising to modulate the created PL properties.

Interestingly, the PL quantum yield (φ) of the SWNT/2DzAr5 was enhanced just by the change in the surfactant from sodium dodecyl sulfate (SDS) to sodium dodecyl benzene sulfonate (SDBS). The relative quantum yields were estimated using Styryl 13 as a reference, which is frequently used for the φ evaluation of SWNT systems^23^. The surfactant exchange reaction was carried out through dialysis of an SDS dispersion of the SWNT/2DzAr5 in an SDBS solution (cut-off Mw = 10,000). As shown in Fig. 3e, significant enhancement of the PL intensity was observed after the dialysis treatment; namely, the φ value of the SWNT/2DzAr5 sample in the SDBS solution was estimated to be 3.15%, which is 1.9 times higher than that in the SDS solution (φ = 1.70%) and 6.2 times higher than that of the pristine SWNTs (φ = 0.507%). Importantly, the *E*_11_^2*^ peak was not evident when the SDBS-dispersed SWNTs were used as the reaction precursor from the beginning, which is probably due to the tight binding of SDBS molecules to the SWNT surfaces that would hamper the reaction of 2DzAr5 with the SWNTs^24^,^25^. Associated with this behavior, the densely-covered structure of SDBS on SWNTs would contribute to the enhanced emission efficiency of SWNT/2DzAr5 through possible isolation of the tubes from the water environment^26^,^27^. This result indicates that the other surfactants such as sodium deoxycholate^28^ and a biocompatible lipid^5^ could modify and enhance the functionalities of the resulting SWNTs by the surfactant exchange reactions. As characteristics of the bisdiazonium-modified SWNTs, the *E*_11_^2^* showed a much greater Stokes shift (~270 nm) than *E*_11_*, and the *E*_11_ excitation at 980 nm provided a higher PL intensity of the *E*_11_^2^* as shown in Fig. 3e. Thus, these features are prominent for advanced bio-imaging and sensing applications with the NIR excitation/emission feature, which could realize versatile tuning of the emission wavelengths in the tissue-penetrable NIR light range and the reduction of background signals including autofluorescence from tissues and Rayleigh scattering from incident lights.

**Figure 4:**
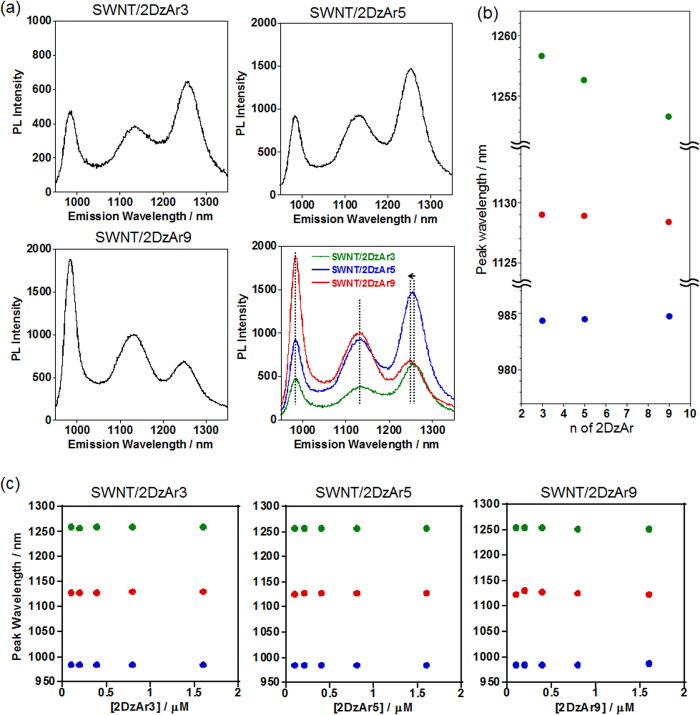
PL properties of the SWNT/2DZArn (n = 3, 5, 9). (**a**) PL spectra of SWNT/2DzArn (n = 3, 5, 9) in D_2_O and their overlaid spectra, in which the observed peak shift for *E*_11_^2^* is denoted with an arrow. [2DzArn] = 0.40 μM. For the deconvoluted spectra, see Fig. S3. (**b**) Peak wavelength shifts of *E*_11_^2^*, *E*_11_*, and *E*_11_ for each SWNT/2DzArn with respect to the methylene spacer lengths of 2DzArn. (**c**) Peak wavelengths (blue: *E*_11_, red: *E*_11_*, green: *E*_11_^2^*) of each SWNT/2DzArn sample as a function of the 2DzArn concentrations in the reactions.

When 2DzAr3 or 2DzAr9 was reacted with the SWNTs instead of 2DrAr5, new peaks assignable to *E*_11_^2*^ are observed in both samples (Figs 4a and S3). Interestingly, the wavelengths of the *E*_11_^2*^ were blue-shifted with the increase in the spacer length of the 2DrArn; namely, the *E*_11_^2*^ peaks were observed at 1258 nm, 1256 nm, and 1253 nm for the SWNT/2DzAr3, SWNT/2DzAr5 and SWNT/2DzAr9 systems, respectively (Fig. 4a,b). As shown in Figs 4c and S4, even if the concentrations of 2DzArn were changed, the observed *E*_11_^2*^ peaks always appeared at the same wavelengths in each SWNT/2DrArn sample with the same spacer length (1258 nm for SWNT/2DzAr3, 1256 nm for SWNT/2DzAr5, 1253 nm for SWNT/2DzAr9). The degree of the 2DzArn-modification was estimated by Raman spectroscopy to investigate the difference in reactivity of the 2DzArns (n = 3, 5 and 9) with the SWNTs. Here, the G/D ratios were used, in which the signals of the G and D bands are related to the crystallinity of the graphitic structures and defect sites on the SWNT, respectively^29^. As shown in Fig. S5, the G/D ratios estimated from the Raman spectra are 15.8, 24.2, 36.8, and 11.8 for the SWNT/2DzAr3, SWNT/2DzAr5, SWNT/2DzAr9, and SWNT/1Dz, respectively, which were lower than that (73.0) of the pristine SWNTs due to the chemical modification of the SWNTs in these samples. Based on the comparison of the obtained G/D ratios, the SWNT/2DrAr9 showed a relatively high value, indicating less *sp*^3^ defect formation. This result is consistent with the fact that SWNT/2DrAr9 has a relatively weaker *E*_11_^2^* than those of SWNT/2DrAr3 and SWNT/2DrAr5 prepared with the same reactant concentrations, as can be seen in Figs 3c and S6. It is suggested that modification of one terminal diazonium (ω-site) group would be somewhat predominant after reaction of the α−terminal diazonium of the 2DrAr3 (or 5) having a shorter spacer. For the reaction using 0.80 μM 2DzAr9, which is twice the concentration used for the sample shown in Fig. 4a, a higher *E*_11_^2^* is observed owing to the increase in the 2DzAr9-modified sites (Fig. S7).

The obtained results reveal that the wavelength of the generated *E*_11_^2*^ does not depend on the degree of the chemical modification, but depends entirely on the chemical structures of the reactants. In addition, the observed noteworthy trend is that the PL peaks are red-shifted when shorter spacer compounds are reacted with the SWNTs, indicating that the expected closer modification of the bisdiazonium groups affects the change in energy level of the reacted sites. Therefore, the spacer length of bisdiazonium compounds is one of the important factors in regulating the energy levels of local-f-SWNTs, due to the local arrangement of the introduced *sp*^3^ defects, as predicted from the simulation results.

## Conclusion

We have demonstrated *E*_11_^2*^ generation from SWNTs, in which new bisdiazonium compounds (2DzArn: n = 3, 5 and 9) were designed and synthesized for the preparation of local-f-SWNTs. When using 2DzArn, we revealed that new PL peaks (*E*_11_^2*^) appeared in the longer wavelength regions compared to that using a monodiazonium modifier (1Dz), and the peak maxima of the *E*_11_^2*^ depended on the spacer length of the 2DzArn. Such a large redshift of *E*_11_^2*^ was in reasonably good agreement with the range of the calculated effect on HOMO-LUMO gap, which is 0.37 eV and 0.21 eV for **R3** (0.58 eV) and **N4** (0.74 eV) doping geometries compared to the calculated bandgap of the pristine tube. The high intensity and relative sharpness of the *E*_11_^2*^ peak confirmed that the doping process is not random; that is, when the first *sp*^3^ center is created, the radical character around the site activates the wall of the carbon nanotubes through delocalization of the unpaired electron. When the other end of the doping molecule is attached, it chooses one of the high spin-density spots, leading to a *sp*^3^ pair with a strong interaction, like **R3** or **N4**.

Basically, the intrinsic fluorescence wavelengths of the pristine SWNTs are dominated by the rolled-up graphene sheet structures that are classified with chiral indices (n,m). Indeed, changes in the microenvironments around the SWNT surfaces (for example, surfactants and solvents) can provide some PL spectral shifts. However, our finding is quite different from such studies because we have achieved the generation of a new PL signal (*E*_11_^2*^) from SWNTs through structural modeling of the emissive doped sites by designing modifier molecules (bisdiazonium). Therefore, the present findings could lead to the SWNT PL modulation based on local covalent modification and are highly important for the fundamental understanding of the intrinsic PL properties of SWNTs as well as exciton engineering–based applications including photonic devices and (bio)imaging/sensing.

## Methods

### Materials

The SWNTs (CoMoCAT (6,5) rich) were purchased from SouthWest Nanotechnologies. Sodium hydrate and sodium nitrite were obtained from Wako Pure Chemical Industries. Sodium dodecyl benzene sulfonate (SDBS), 4-hydroxyacetanilide, 1,3-dibromopropane and 1,9-dibromononan were purchased from the Tokyo Chemical Industry Co. Tetrafluoroboric acid (48 wt.% aqueous solution) and 4-methoxybenzenediazonium tetrafluoroborate were purchased from the Sigma-Aldrich Co. D_2_O, potassium hydroxide, 1,5- bis(4-aminophenoxy)pentane and Styryl 13 were obtained from Cambridge Isotope Laboratories, Kishida Chemical Co., Angene International, and EXCITON Inc., respectively. All chemicals were used as received.

### Instruments

The ^1^H and ^13^C NMR spectra were recorded using a Bruker AV300m spectrometer (300 MHz). High-resolution electrospray mass measurements (HR-ESI-MS) were carried out using a Bruker MicroTOF-QIII electrospray ionization time-of-flight (ESI-TOF) mass spectrometer. The UV/vis/NIR and PL together with the 2D PL mapping spectra were measured using a V-670 (JASCO) and a HOLIBA JOBIN YVON spectrofluorometer (FluorologR-3 with FluorEssence), respectively. The Raman spectra at an excitation of λ = 633 nm were recorded by a RAMANtouch spectrometer (Nanophoton Corporation). Quartz cells with a 1-cm path length were used for the optical measurements. MilliQ water was prepared by an ultrapure water system equipped with an Elix-5 kit (Millipore Co.). For preparation of the SWNT dispersions, a bath-type sonicator (AS ONE, US-1R), a tip-type sonicator (MISONIX, XL-2000) and an ultracentrifuge (Hitachi, himac CS 100 GXL) were used.

### Synthesis

The 2DzArns were synthesized by the following 3 steps: (i) the Williamson ether synthesis using 4-hydroxyacetanilide and dibromoalkanes with different chain length n = 3, 5 or 9, (ii) hydrolysis of the amide group for the deprotection of the amine group and (iii) diazotization. Syntheses of all the 2DzArn compounds were carried out under the same reaction conditions except for the column chromatography purification procedure using silica gel and the eluent solvent of CH_3_OH:CHCl_3_ = 1:9 v/v only for the first etherification step of the 2DrAr9 synthesis. Therefore, as a representative result, the synthetic details for 2DzAr3 are described below and the characterization data for 2DzAr5 and 2DzAr9 are shown at the end of this synthesis section.

#### N,N’-((Propane-1,3-diylbis(oxy))bis(4,1-phenylene))diacetamide (**1**)

In a 25 mL two-necked flask, 0.400 g of NaH (40% oil, 10 mmol) was dispersed in 5 mL of anhydrous DMF with cooling in an ice bath and 4-hydroxyacetanilide (1.517 g, 10 mmol) in 5 mL of anhydrous DMF and 1.3 dibromopropane (0.51 mL, 5 mmol) were then added. After stirring while cooling in an ice bath for 8 h, the reaction mixture was filtered and the filtrate was poured into water. The generated precipitate was then collected by filtration and dried in vacuo to provide a colorless powder (0.65 g, yield 38%)^30^,^31^. ^1^H NMR (300 MHz, DMSO-d_6_) *δ*/ppm = 9.77 (s, 2H; 2NH), 7.46 (d, *J* = 9.1 Hz, 4H; Ar), 6.87 (d, *J* = 9.1 Hz, 4H; Ar), 4.06 (t, *J* = 6.3 Hz, 4H; 2OCH_2_), 2.12 (quintet, *J* = 6.4 Hz, 2H; OCH_2_*CH*_*2*_), 1.99 (s, 6H, 2CH_3_); HR-ESI-MS *m/z* calcd. for [M + Na]^+^ = C_19_H_22_N_2_NaO_4_^+^: 365.1472 [*M*], found: 365.1477.

#### 4,4′-(Propane-1,3-diylbis(oxy))dianiline (**2**)

In a 25 mL two-necked flask, compound **1** was dissolved in 15 mL of ethanol to which a 20 M KOH solution (6 mL) was added. The mixed solution was refluxed for 8 h, then poured into water to generate a precipitate, which was collected and dried in vacuo, providing a pale yellow solid as the product (305 mg, yield 78%)^30^,^31^ ^1^H NMR (300 MHz, CDCl_3_): *δ*/ppm = 6.75 (d, *J* = 8.9 Hz, 4H; Ar), 6.63 (d, *J* = 8.9 Hz, 4H; Ar), 4.07 (t, *J* = 6.2 Hz, 4H; 2OCH_2_), 3.41 (brs, 4H; 2NH_2_), 2.18 (quintet, *J* = 6.2 Hz, 2H; OCH_2_*CH*_*2*_) HR-ESI-MS: *m/z* calcd for [M + Na]^+^ = C_15_H_18_N_2_NaO_2_^+^: 281.1260, found: 281.1257; Elemental analysis (%): calcd for C_15_H_18_N_2_O_2_: C 69.74, H 7.02, N 10.84; found C 69.83, H 7.10, N 10.64.

#### 2DzAr3

To a 5 mL two-necked flask, aqueous tetrafluoroboric acid (48 wt.%, 520 μL) and MilliQ water (600 μL) were added followed by cooling in an ice bath, into which compound **2** (30.9 mg, 0.12 mmol) was dissolved. An aqueous solution of sodium nitrite (0.48 mmol) was dropwise added and the reaction mixture was stirred for 20 min. The resulting solid was collected and washed with diethyl ether, then dried in vacuo to provide a pale red solid (43 mg, yield 79%).^1^H NMR (300 MHz, CD_3_CN): *δ*/ppm = 8.38 (d, *J* = 9.5 Hz, 4H; Ar), 7.31 (d, *J* = 9.5 Hz, 4H; Ar), 4.48 (t, *J* = 6.0 Hz, 4H; 2OCH_2_), 2.40 (quintet, *J* = 6.0 Hz, 2H; OCH_2_*CH*_*2*_), ^13^C NMR (75 MHz, CD_3_CN): *δ*/ppm = 170.34, 136.81, 119.17, 102.79, 67.87, 28.73, ^19^F NMR (282 MHz, CD_3_CN): *δ*/ppm = −151.71; HR-ESI-MS : *m/z* calcd for the cationic moiety [*M*]^2+^ = C_15_H_14_N_4_O_2_^2+^: 141.0559, found: 141.0542.

#### 2DzAr5

^1^H NMR (300 MHz, CD_3_CN): *δ*/ppm = 8.37 (d, *J* = 9.6 Hz, 4H; Ar), 7.33 (d, *J* = 9.6 Hz, 4H; Ar), 4.30 (t, *J* = 6.4 Hz, 4H; 2OCH_2_), 1.60–1.75 (m, 6H; 3CH_2_); ^13^C NMR (75 MHz, CD_3_CN): *δ*/ppm = 170.81, 136.75, 119.19, 102.20, 71.54, 28.83, 22.63; ^19^F NMR (282 MHz, CD_3_CN): *δ*/ppm = −151.80 ppm; HR-ESI): *m/z* calcd for cationic moiety of [*M*]^2+^ = C_17_H_18_N_4_O_2_^2+^: 155.0715; found: 155.0717.

#### 2DzAr9

^1^H NMR (300 MHz, CD_3_CN): *δ*/ppm = 8.37 (d, *J* = 9.6 Hz, 4H; Ar), 7.33 (d, *J* = 9.6 Hz, 4H; Ar), 4.30 (t, *J* = 6.4 Hz, 4H; 2OCH_2_), 1.60–1.75 (m, 6H; 3CH_2_); ^13^C NMR (75 MHz, CD_3_CN): *δ*/ppm = 169.93, 135.73, 118.20, 101.01, 70.86, 29.10, 28.87, 28.27, 25.34; ^19^F NMR (282 MHz, CD_3_CN): *δ*/ppm = −151.86; HR-ESI-MS: *m/z* calcd for the cationic moiety of [*M*]^2+^ = C_21_H_26_N_4_O_2_^2+^: 183.1028, found: 183.1037.

### Preparation of local-f-SWNTs using diazonium compounds

The SWNTs and SDS were dried at 120 °C for 2 h. In a 50 mL glass bottle, 5 mg of the SWNTs was dispersed in a D_2_O solution of SDS (2 wt.%) and sonicated using a bath-type sonicator for 1 h and using a tip-type sonicator for 30 min. The resulting dispersion was supercentrifuged at 150,000 g for 4 h and the supernatant was collected as a SWNT dispersion. The obtained SWNT dispersions were diluted with D_2_O to prepare the appropriate concentrations for absorption and PL measurements, by which the measurement samples were SWNTs dispersed in 0.2 wt.% SDS solutions.

The reactions were carried out by mixing the SWNT dispersion and solutions of the diazonium compounds in the dark for 10 days^14^. All reactions and measurements were performed at room temperature controlled around 20 °C.

### Quantum yield estimation

The relative quantum yield using Styryl 13 as a reference was estimated based on the following equation^23^:


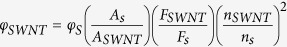


where φ is the quantum yield, A is absorbance, F is the integrated area of the PL, n is the refractive index of the solvents, and the subscripts of SWNT and S represent the SWNTs and solvents, respectively.

### Calculations

The models used for the calculations for the (6,5) SWNTs were infinite (3D periodic) with a regular unit cell of approximately 4 nm length along the *z* axis. To avoid any inter-tube interactions, the separation between the tubes in the *x* and *y* directions were at least 4 nm. While the SWNTs have a finite size in reality, the infinite models help to avoid edge effects typical to short, finite length tube models, and therefore they can provide more realistic electronic structures and band gaps.

The calculations of the geometry, energy and electronic structure of the unit cells were performed using the SCC-DFTB method^32^ with the DFTB+ program (ver. 1.2)^33^ and the “mio” Slater-Koster set^32^. DFTB (Density Functional Tight Binding) is a semi-empirical method with accuracy close to the DFT methods, which we have successfully used before to describe nanocarbon systems^18^.

The calculations were performed with a 1 × 1 × 2 Monkhorst–Pack grid^34^. Calculations with the 1 × 1 × 4 grid showed no significant improvement in the energies and band gaps. The geometries were optimized with the convergence criteria of 5 meV. The electronic converging criterion was 0.27 eV for the SCF cycles. Due to the small band gap, we used a small Fermi broadening with T = 100 K. Broyden mixing was applied using the mixing parameter of 0.2^35^. The edges of the conductance and valence bands were extracted from the calculated band structures, and were referred to as the LUMO and HOMO levels, respectively, for simplicity.

## Additional Information

**How to cite this article**: Shiraki, T. *et al*. Emergence of new red-shifted carbon nanotube photoluminescence based on proximal doped-site design. *Sci. Rep*. **6**, 28393; doi: 10.1038/srep28393 (2016).

## Supplementary Material

Supplementary Information

## Figures and Tables

**Table 1:** Calculated properties of the doped geometries.

	D	Z1	Z2	Z3	Z4	R1	R2	R3	R4	N1	N2	N3	N4
a) HOMO-LUMO gap (eV)	0.00	0.94	0.03	0.02	0.00	0.95	0.01	0.58	0.00	0.01	0.95	0.01	0.74
b) Spin density	0.00	0.12	−0.02	0.00	0.00	0.13	−0.01	0.02	0.00	−0.02	0.11	−0.01	0.04
c) Energy (kcal/mol)	0.00	−29.2	−0.95	−0.02	0.69	−27.4	4.72	−12.0	0.28	5.52	−27.4	2.33	−15.2

(a) HOMO-LUMO gap, (b) spin-density for single doped geometries, and (c) stability compared to the D-doped site. Capital letters correspond to the doped positions shown in Fig. 1.
