# Supplementary material for: Emergence of new red-shifted carbon nanotube photoluminescence based on proximal doped-site design
Source: Sci Rep. 2016 Jun 27;6:28393. doi: 10.1038/srep28393 (PMC4921849; doi:10.1038/srep28393)
Supplement: Supplementary Information [file srep28393-s1.pdf]

Supplementary Information

Emergence of new red-shifted carbon nanotube photoluminescence based on proximal doped-site design

Tomohiro Shiraki[*1,2], Tomonari Shiraishi[1], Gergely Juhász[3] & Naotoshi Nakashima[*1,2]

[1] Department of Applied Chemistry, Graduate School of Engineering, Kyushu University, 744 Motooka, Nishi-ku, Fukuoka 819-0395, Japan. [2] International Institute for Carbon-Neutral Energy Research (WPI-I2CNER), Kyushu University, 744 Motooka, Nishi-ku, Fukuoka, Japan. [3] Department of Chemistry, Graduate School of Science, Tokyo Institute of Technology, Ookayama, Meguro-ku, Tokyo, 152-8550, Japan.

Correspondence and requests for materials should be addressed to T.S. (email: shiraki.tomohiro.992@m.kyushu-u.ac.jp) or N.N. (email: nakashima.naotoshi.614@m.kyushu-u.ac.jp).

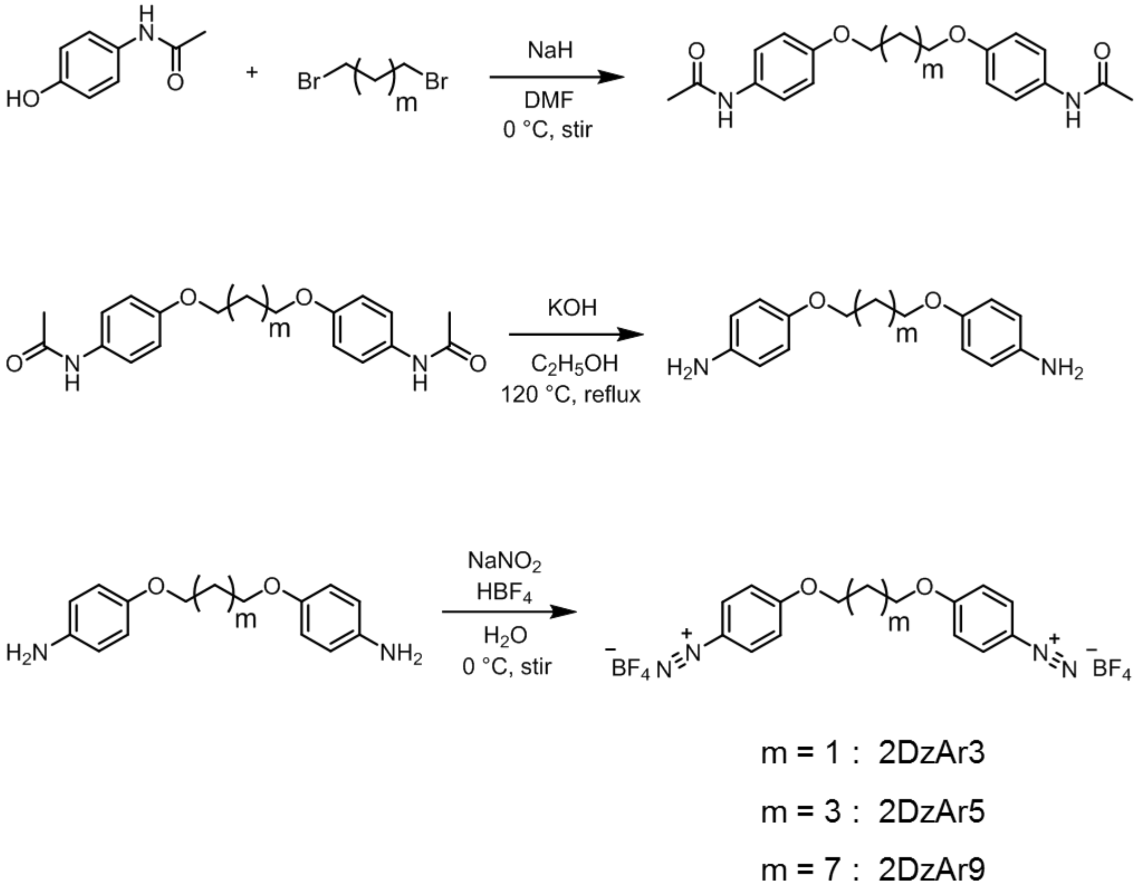

Figure S1. Synthetic routes of the 2DzArn (n = 3, 5 and 9).

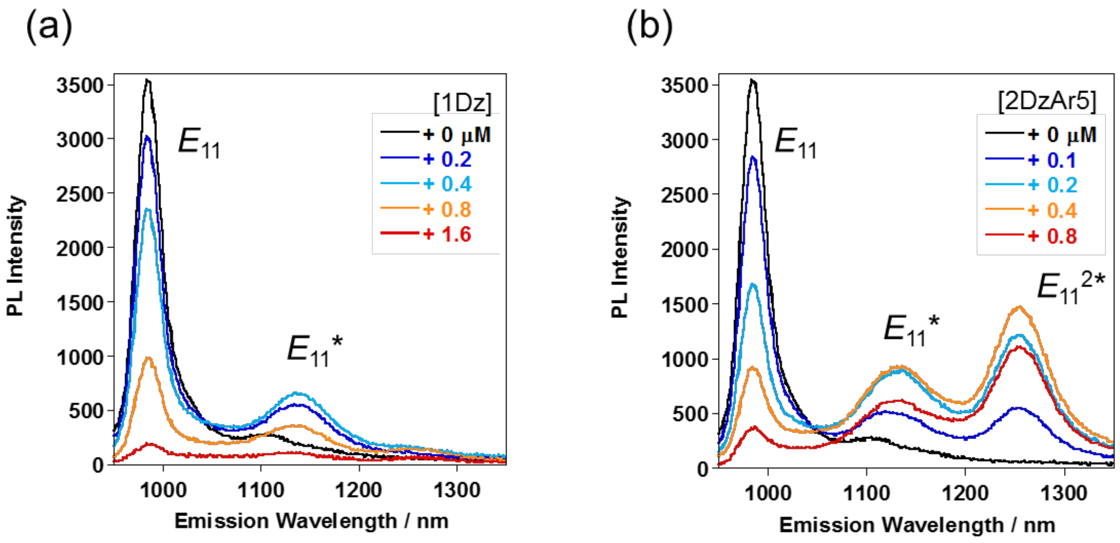

Figure S2. PL spectral changes of (a) SWNT/1Dz and (b) SWNT/2DzAr5 with respect to concentrations of the added diazonium compounds.

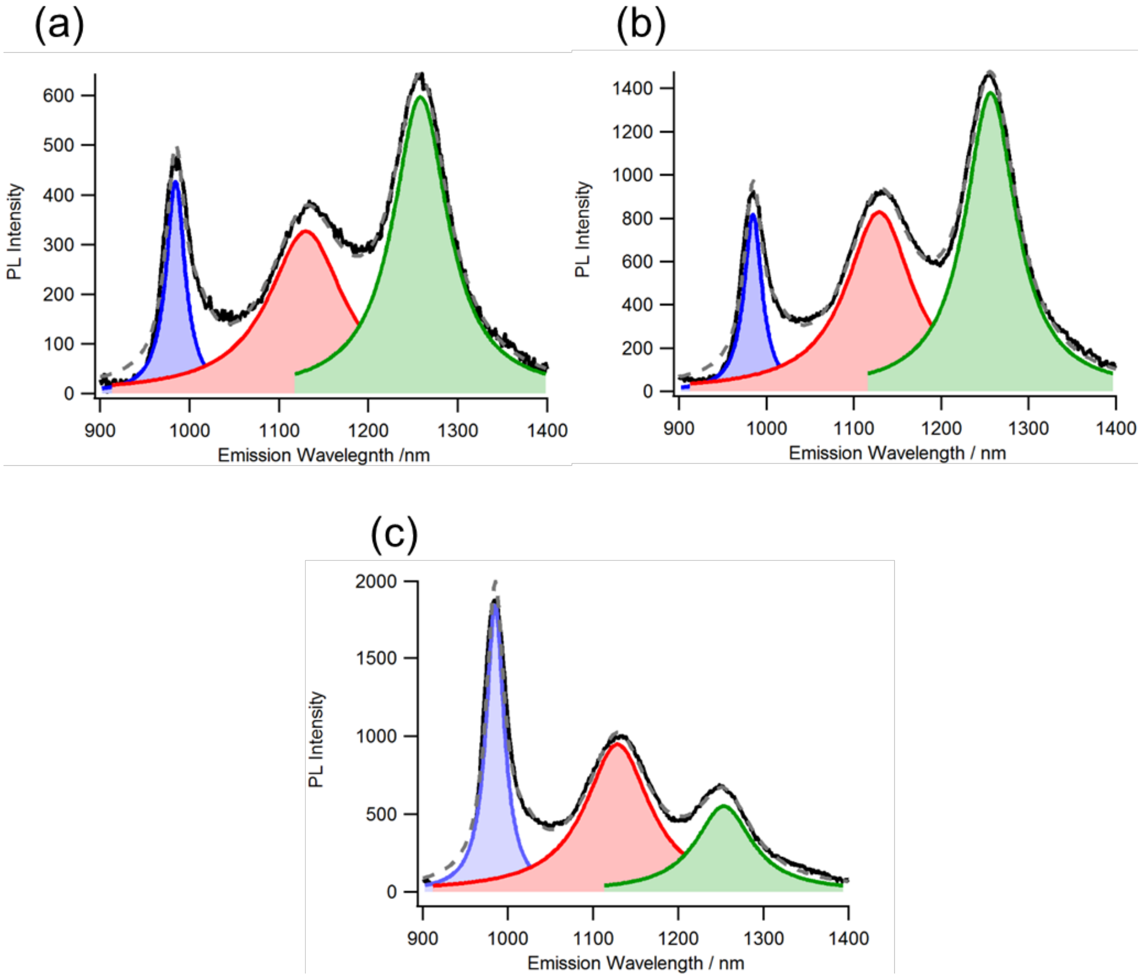

Figure S3. Deconvoluted PL spectra of (a) SWNT/2DzAr3, (b) SWNT/2DzAr5 and (c) SWNT/2DzAr9 in D_2O. [2DzArn] = 0.4 μM.

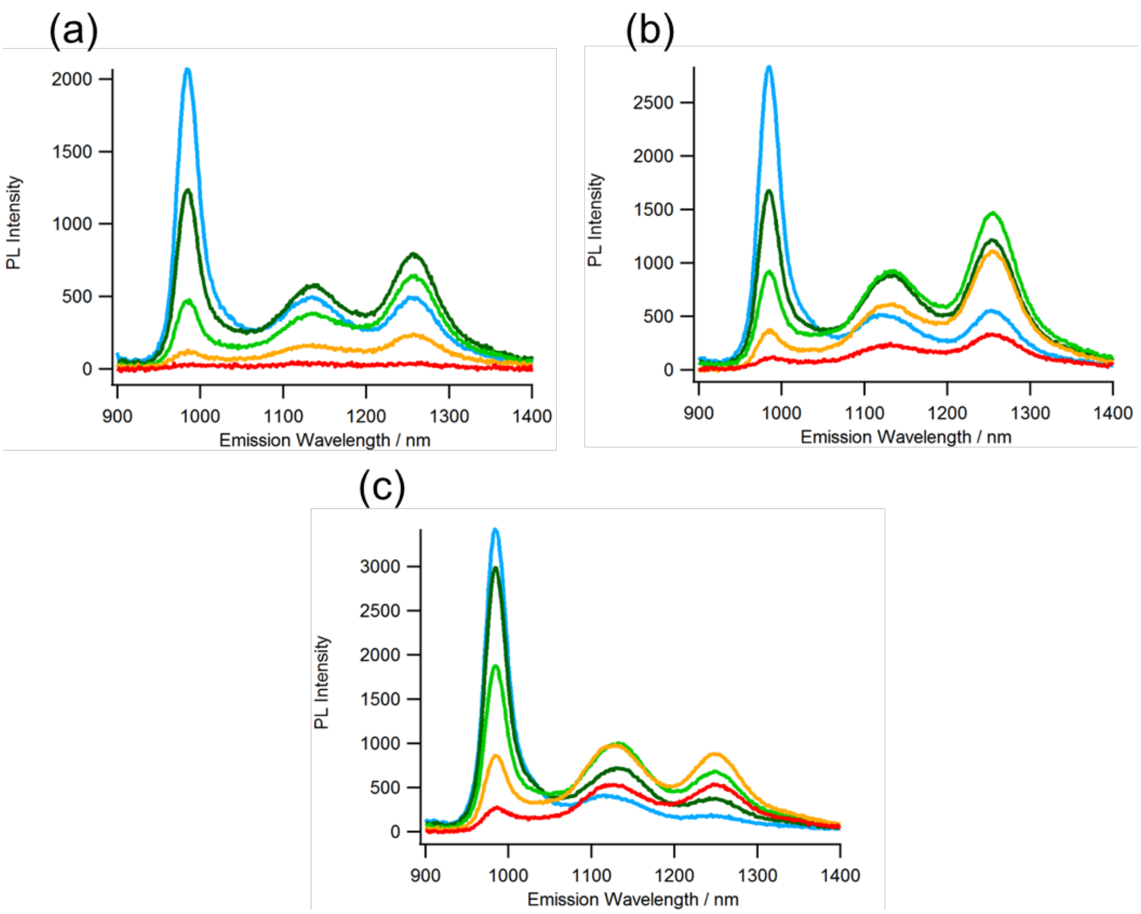

Figure S4. PL spectra of (a) SWNT/2DzAr3, (b) SWNT/2DzAr5 and (c) SWNT/2DzAr9 prepared by using different concentrations of 2DzArn: [2DzArn] = 0.10 (light blue), 0.20 (dark green), 0.40 (bright green), 0.80 (orange) and 1.6 μM (red).

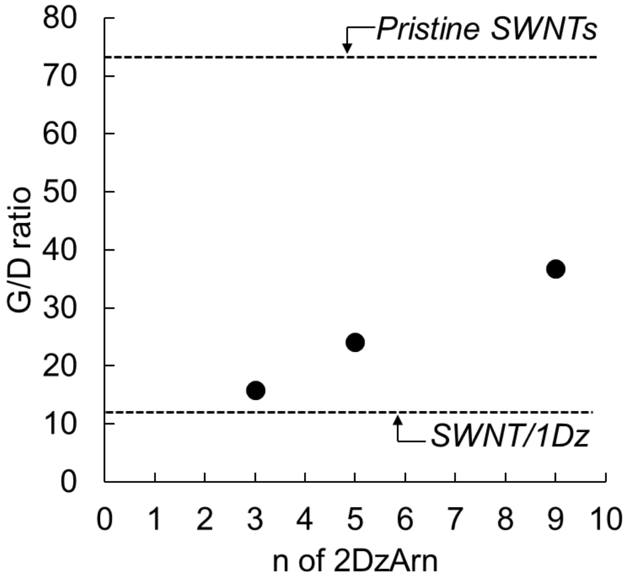

Figure S5. G/D ratios of SWNT/2DzArn, SWNT/1Dz and pristine SWNTs. The laser light with 570 nm was utilized for excitation and was irradiated to the D_2O dispersion samples. [2DzArn] = 0.4 μM and [1Dz] = 0.8 μM was used for chemical modification to prepare the same concentrations based on the diazonium group.

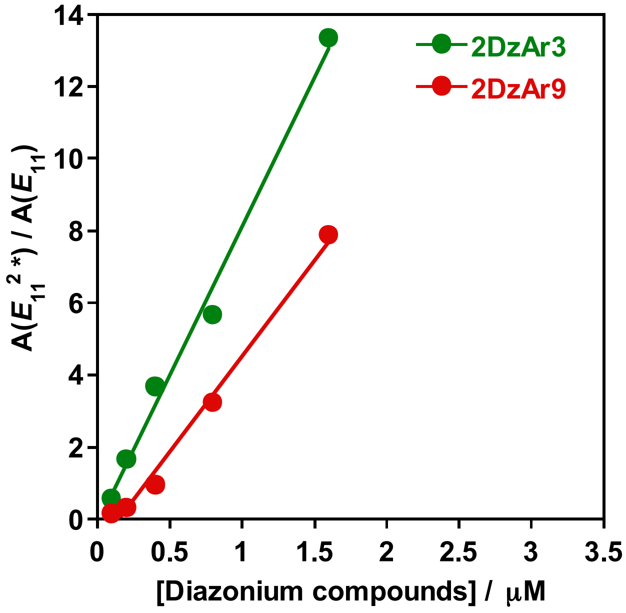

Figure S6. Integrated area ratio of $E_{11}{}^{2}*$ to $E_{11}*$ as a function of the diazonium concentrations for SWNT/2DzAr3 (green dot) and SWNT/2DzAr9 (red dot).

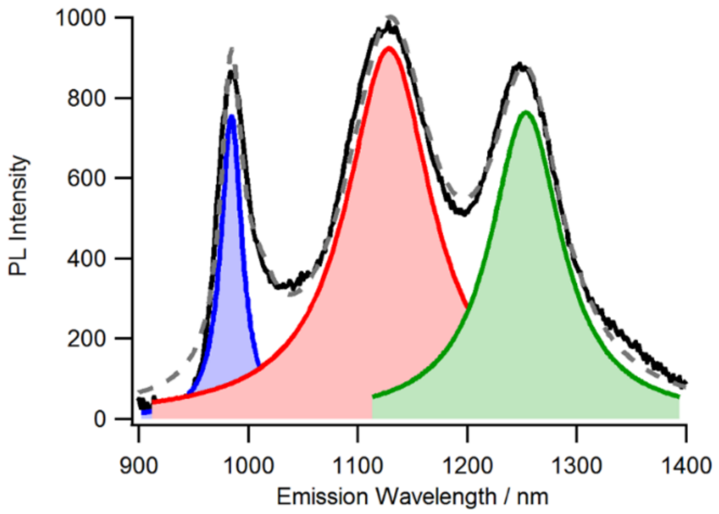

Figure S7. PL spectrum of SWNT/2DzAr9 prepared by using 0.80 μM 2DzAr9.
